# Three-dimensional Echocardiography for Percutaneous Occlusion of Patent Foramen Ovale

**DOI:** 10.5935/abc.20160036

**Published:** 2016-03

**Authors:** Robinson Poffo, Paola Keese Montanhesi, Renato Bastos Pope, Alisson Parrilha Toschi, Marcelo Campos Vieira

**Affiliations:** Hospital Israelita Albert Einstein − São Paulo, SP - Brazil

**Keywords:** Foramen Ovale, Patent / surgery, Echocardiography, Three-Dimensional, Minimally Invasive Surgical Procedures

Female patient, 31 years old, with a history of ischemic stroke 7 years before, was referred for treatment of Patent Foramen Ovale (PFO). She only had a complaint of migraine with aura, with no focal neurological abnormalities.

On July 2, 2015, she was submitted to an uneventful percutaneous PFO occlusion using a Cardia Intrasept^TM^ prosthesis (CARDIA, Inc., Burnsville, MN, USA). Intraoperative three-dimensional transesophageal echocardiography confirmed correct prosthesis positioning and absence of residual shunt through the bubble test. Current guidelines of the two most important international societies of echocardiography, the European Association of Cardiovascular Imaging and the American Society of Echocardiography, determine that the three-dimensional echocardiography is the echocardiographic modality of choice for monitoring, following and guiding percutaneous procedures performed in hemodynamics and hybrid rooms, as in this case.^1^

The patient was discharged on the first postoperative day. After 30 days, adequate placement of the device was confirmed with PFO occlusion through transthoracic echocardiography. The patient remains in outpatient care, with migraine improvement.

**Figure 1:**
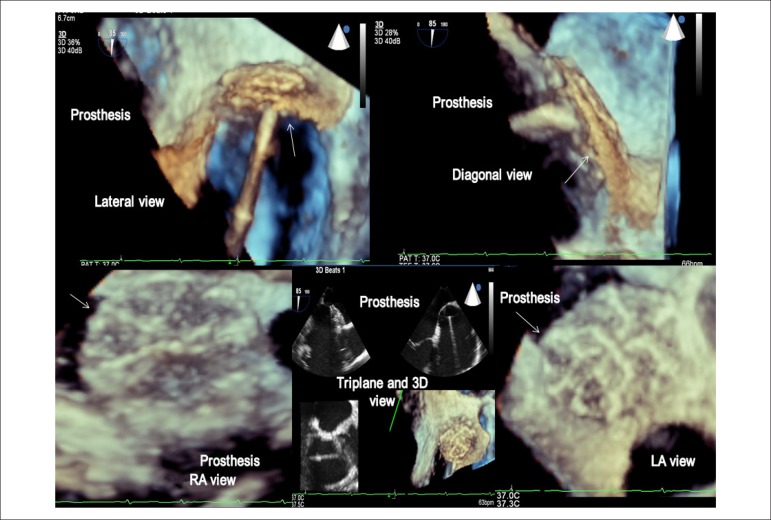
Three-dimensional transesophageal echocardiography image in multiple planes (from the left atrium, the right atrium, from the lateral, diagonal and triplane views) of the Cardia Intrasept^TM^ prosthesis (arrow) well positioned in the atrial septum in a patient with patent foramen ovale. RA: right atrium; LA: left atrium.
